# Thermal annealing effects on tunnel oxide passivated hole contacts for high-efficiency crystalline silicon solar cells

**DOI:** 10.1038/s41598-022-18910-5

**Published:** 2022-09-02

**Authors:** Yong-Jin Kim, I Se Kweon, Kwan Hong Min, Sang Hee Lee, Sungjin Choi, Kyung Taek Jeong, Sungeun Park, Hee-eun Song, Min Gu Kang, Ka-Hyun Kim

**Affiliations:** 1 Photovoltaics Research Department, Korea Institute of Energy Research, Daejeon, 34129 South Korea; 2 Department of Physics, Chungbuk National University, Cheongju, Chungbuk 28644 South Korea

**Keywords:** Solar cells, Electronic devices

## Abstract

Tunnel oxide passivated contacts (TOPCon) embedding a thin oxide layer between polysilicon and base crystalline silicon have shown great potential in the development of solar cells with high conversion efficiency. In this study, we investigate the formation mechanism of hole-carrier selective contacts with TOPCon structure on n-type crystalline silicon wafers. We explore the thermal annealing effects on the passivation properties in terms of the stability of the thermally-formed silicon oxide layer and the deposition conditions of boron-doped polysilicon. To understand the underlying principle of the passivation properties, the active dopant in-diffusion profiles following the thermal annealing are investigated, combined with an analysis of the microscopic structure. Based on PC1D simulation, we find that shallow in-diffusion of boron across a robust tunnel oxide forms a p–n junction and improves the passivation properties. Our findings can provide a pathway to understanding and designing high-quality hole-selective contacts based on the TOPCon structure for the development of highly efficient crystalline silicon solar cells.

## Introduction

To achieve high conversion efficiency in crystalline silicon (c-Si) solar cells, the carrier selective contacts are of considerable importance in suppressing the recombination current (*J*_0_) of the minority carriers and gaining a high open-circuit voltage (*V*_OC_)^1–8^. Among a wide range of approaches, the tunnel oxide passivated contacts (TOPCon) are promising candidates because of their low recombination loss and high carrier selectivity with a high conversion efficiency of 25.7%^5^. The TOPCon structure, which introduces a buffer layer of tunnel oxide between heavily-doped polysilicon (poly-Si) and the base wafer, exhibits high-quality passivation owing to an indirect metal contact with the c-Si wafer, one-dimensional current flow, and tunneling-based carrier selectivity^2–8^.

In the case of the electron-selective TOPCon, it was proposed that the different band offsets between Si and SiO_2_ (4.7 eV for the valence band and 3.2 eV for the conduction band) offer asymmetric tunnel barriers for the electrons and holes, which provide effective electron-selective contacts^9,10^. However, it has been reported that the tunnel mechanism is not the only working principle in the TOPCon structure; conduction via pinholes or/and nanopits within the oxide layer must also be considered^10–13^. Furthermore, for better carrier transport, post-deposition annealing at an optimal temperature is required to promote the moderate pinhole/nanopit density and to reduce the interface state density induced by the saturation of SiO_*x*_^6,11–16^. If the annealing temperature is excessively high, the tunnel oxide is no longer stable because of the formation of highly dense pinholes/nanopits^11–16^ and introduction of redundant dopant in-diffusion into the c-Si substrate^3,4,17,18^. The latter increases the recombination loss of the minority carriers owing to the Auger recombination rate and deficient electronic band bending^3,4,17,18^.

In contrast to the electron-selective TOPCon, studies on hole-selective contacts are limited, and the passivation quality is relatively poorer than that of the electron counterpart due to the recombination loss via B-O complex defects^2,19–31^. Although the traditional tunneling-based interpretation indicates that the hole-selective contacts seem to be inefficient, studies have reported on their high implied *V*_OC_ (i*V*_OC_) of approximately 720 mV and low recombination current that is less than 10 fA cm^-2^^21,23,25^. For example, the replacement of B-doped poly-Si with Ga-doped poly-Si prevents the formation of the B-O complex defects with the achievement of a high i*V*_OC_ of 731 mV^21^. The substitution of AlO_*x*_ to SiO_*x*_ achieved an i*V*_OC_ of 723 mV and *J*_0_ of 6.6 fA cm^−2^ due to the high negative space charge density of AlO_*x*_^25^. Similar to the electron-selective contacts, the hole-selective contacts require the stability of the tunnel oxide layer for chemical passivation and shallow in-diffusion for field-effect passivation^30,31^. However, the underlying formation mechanism of the hole-carrier selectivity via thermal annealing and the microscopic structure have not yet been satisfactorily explained. In these regard, we investigated the passivation properties of the hole-selective contacts based on the TOPCon structure under thermal annealing. We examined the microstructural evolution to directly observe the annealing effect on the tunnel oxide and passivation under different deposition conditions of the B-doped poly-Si. Active boron in-diffusion profiles were studied to understand the influence on the electronic band structure under thermal annealing combined with PC1D simulation. Our studies reveal that the structural stability of the tunnel oxide and the p–n junction formed across the tunnel oxide are essential to improve the passivation properties.

## Results and discussion

### Effect of thermal annealing on passivation

**Figure 1:**
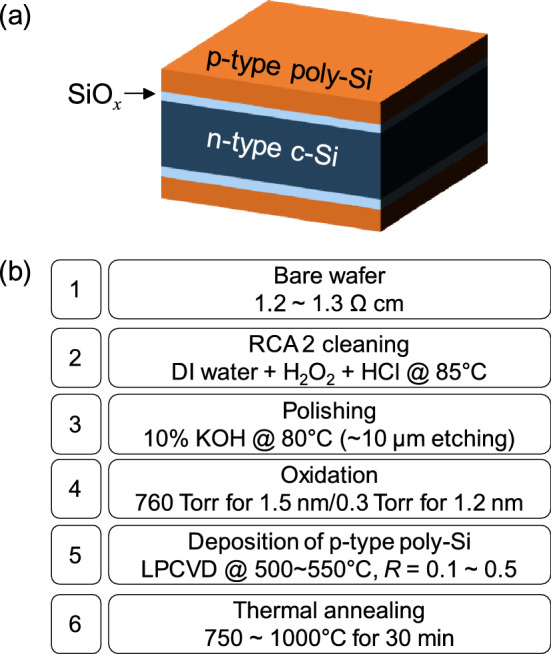
(**a**) Schematic of the symmetric SiO_*x*_/poly-Si stack structure of the hole-selective contacts used in our experiments and (**b**) the fabrication process.

We prepared the tunnel oxide passivated hole contacts as a symmetric structure on an n-type c-Si, as shown in Fig. 1a. The thermal oxidation process was used to induce a silicon oxide (SiO_*x*_) layer as a nominal tunneling barrier. Subsequently, B-doped poly-Si was deposited using the low-pressure chemical vapor deposition (LPCVD) method to ensure a blister-free contact^32–34^. The detailed sample preparation procedures are illustrated in Fig. 1b and described in the section “Methods”.

To assess the thermal stability of the tunnel oxide, we prepared two groups of samples with 1.2-nm-thick and 1.5-nm-thick SiO_*x*_ layers while the p-type poly-Si was deposited at a temperature of 510 °C and gas flow rate ratio (*R*) of 0.5 (where *R* ≡ [B_2_H_6_]/[SiH_4_] in a mixture of diborane (B_2_H_6_) and silane (SiH_4_) precursor gases). Subsequently, the samples were annealed at various temperatures in the range of 750–1000 °C under a nitrogen ambient environment for 30 min. To examine the passivation properties, we extracted i*V*_OC_ and *J*_0_ using the quasi-steady-state photoconductance (QSSPC) method.

**Figure 2:**
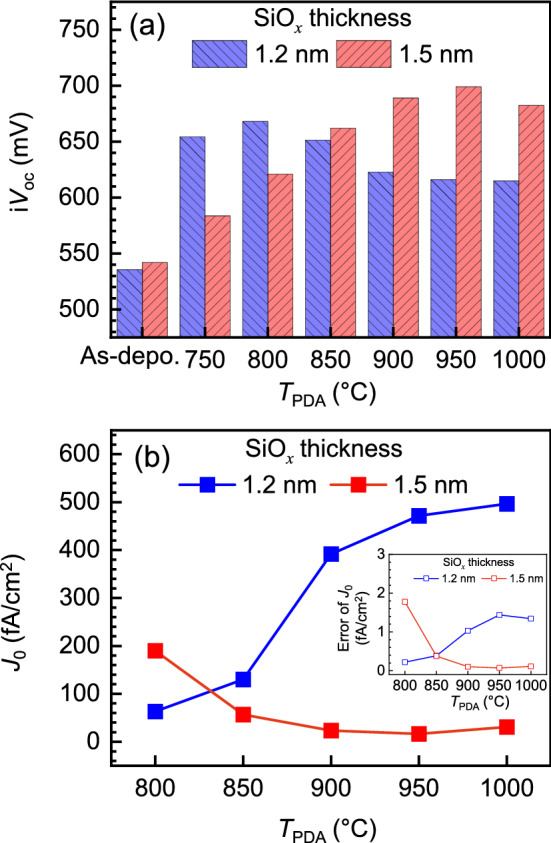
Passivation properties under post-deposition annealing. The measured (**a**) i*V*_OC_ and (**b**) *J*_0_ values after thermal annealing for the samples with 1.2-nm-thick and 1.5-nm-thick SiO_*x*_ layers. The inset of (**b**) indicates errors of *J*_0_ values representing the standard errors of the fittings.

Figure 2a shows the variation of i*V*_OC_ with respect to the post-deposition annealing temperatures (*T*_PDA_) for different thicknesses of the SiO_*x*_ layers*.* Compared to the as-deposited samples, the passivation characteristics under the thermal annealing process showed an improvement in i*V*_OC_ for all samples. This is attributed to the enhanced crystallinity of the poly-Si layer and field-effect passivation by the dopant in-diffusion and activation as well as the reduced interface state density^2,14,17,18,30,31^. For both groups of samples, as *T*_PDA_ was increased, i*V*_OC_ increased and reached the maximum; subsequently, i*V*_OC_ decreased for higher *T*_PDA_. The maximum i*V*_OC_ was obtained at 800 °C for the samples with the 1.2-nm-thick SiO_*x*_ layer and at 950 °C for the samples with the 1.5-nm-thick SiO_*x*_ layer. The relevant opposite trends could be observed for *J*_0_ for both samples. For the samples with the 1.2-nm-thick SiO_*x*_, *J*_0_ was higher for *T*_PDA_ ≥ 800 °C whereas, for the samples with the 1.5-nm-thick SiO_*x*_, the *J*_0_ was minimum at *T*_PDA_ = 950 °C and increased slightly at *T*_PDA_ = 1000 °C. We could not obtain *J*_0_ for *T*_PDA_ < 800 °C due to high fitting errors by low lifetime of the minority carriers (Fig. S1).

### Thermal stability of tunnel oxide

**Figure 3:**
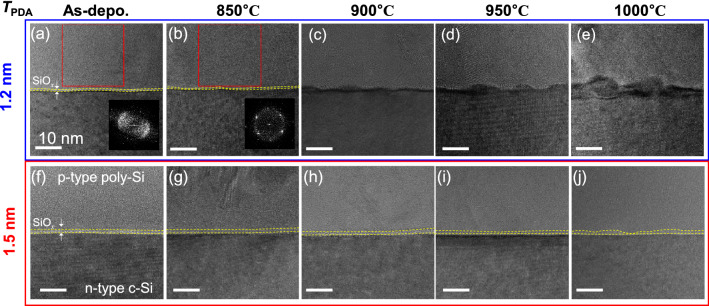
TEM images of the cross-sectional structures around the SiO_*x*_ layers in as-deposited states, annealed at 850 °C, 900 °C, 950 °C, and 1000 °C for the samples with (**a**)–(**e**) 1.2-nm-thick SiO_*x*_ layers and (**f**)–(**j**) 1.5-nm-thick SiO_*x*_ layers. The FFT images in (**a**) and (**b**) were obtained from the red-boxed regions.

To correlate the observed passivation properties to the microstructure of the SiO_*x*_ layers, we obtained cross-sectional transmission electron microscope (TEM) images of the interfacial regions of the poly-Si and c-Si, as shown in Fig. 3. The top and bottom sides of each image correspond to the poly-Si layer and c-Si wafer region, respectively, and the thin bright layer between the poly-Si and wafer corresponds to the thermally grown SiO_*x*_ layer. In the as-deposited samples, the SiO_*x*_ layers had a nearly uniform interface with approximately homogeneous thicknesses; thus, the thicknesses of the SiO_*x*_ layers could be estimated. The normally indicated ‘poly-Si layer’ appeared to be in amorphous phase, as confirmed in a fast Fourier transformed (FFT) image of the red-boxed area in the inset of Fig. 3a. After the thermal annealing, the poly-Si layers showed better crystallinity, as confirmed with the clear diffraction patterns in the FFT image of Fig. 3b, which would improve the passivation properties compared to that of the as-deposited states.

Notably, the degradation of the oxide layer was clearly observable for the sample with the 1.2-nm-thick SiO_*x*_ layer, where the bright oxide layer modulated, and was disrupted for the samples annealed at *T*_PDA_ = 900 °C. For the sample annealed at *T*_PDA_ ≥ 950 °C, the oxide layer had severe damages and an irregular morphology. Meanwhile, a uniform and robust 1.5-nm-thick SiO_x_ layer was observed except at *T*_PDA_ ≥ 950 °C, where the thickness of the SiO_*x*_ layer was partially reduced and inhomogeneous in accordance with our previous study^15^. Thickness reduction can be ascribed to the production of a volatile SiO phase through the annealing process at high temperature in a nitrogen environment produced by the reaction process of SiO_2_ (s) + Si (s) → 2 SiO (g), where s and g denote the solid and gaseous phases, respectively^35,36^. In addition, the growth of the voids/pinholes formed and epitaxial recrystallization of the poly-Si induce the modulation of morphology with the increase of interface defect density by the direct contacts between poly-Si and c-Si^37,38^. Considering the different thicknesses and expected higher oxygen stoichiometry for the thicker SiO_*x*_ layers, a relatively robust SiO_*x*_ layer could be observed for the 1.5-nm-thick layer since the nascent void formation rate for the 1.5-nm-thick SiO_*x*_ layer was slower than that of the 1.2-nm-thick layer. Further studies will be required to quantify the void/pinhole/nanopit density.

The results of the microstructural analysis were consistent with macroscopic passivation properties observed in Fig. 2, which led us to the conclusion that the structural instability deteriorated the passivation characteristics showing the increase of *J*_0_ and decrease of i*V*_OC_. Therefore, optimal thickness is required considering thermal stability and carrier transport through oxide layers^14^.

### Effect of thermal annealing on passivation under different poly-Si growth conditions

In order to optimize the hole-selective contacts and investigate the effects of thermal annealing, we explored the passivation properties with respect to the deposition conditions while maintaining the thickness of the SiO_*x*_ layers at 1.5 nm. We first modulated the gas flow rate ratio (*R*) to fabricate the poly-Si layers at a growth temperature (*T*_G_) of 530 °C using the LPCVD method and annealed the samples at a temperature range of 750–1000 °C. Because a higher *R* corresponds to a higher amount of boron involved during the deposition, higher boron concentration in the poly-Si was expected and reflected in the decrease of sheet resistance (Fig. S2). Subsequently, we extracted the i*V*_OC_ to test the passivation quality of each sample.

**Figure 4:**
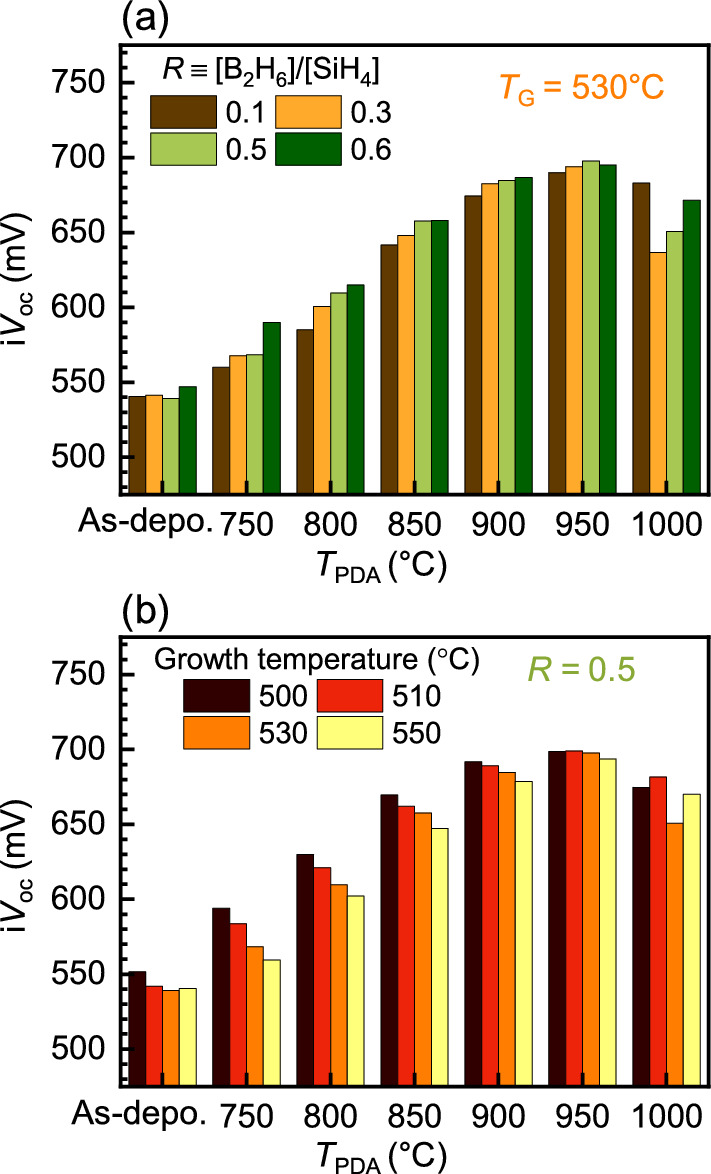
Thermal annealing effect on passivation at different poly-Si growth conditions. Measured i*V*_OC_ values in as-deposited states and after thermal annealing based on the (**a**) flow rate ratio of B_2_H_6_ and SiH_4_ precursor gases and (**b**) growing temperature dependence.

Figure 4a shows the extracted i*V*_OC_ for the samples with different *R*. As discussed before, thermal annealing improved the passivation quality for all samples. For *T*_PDA_ < 950 °C, the annealed samples grown under higher *R* had higher passivation properties. Meanwhile, the improvement of passivation by the higher *R* slowed down as *T*_PDA_ was further increased. Furthermore, at *T*_PDA_ = 950 °C, i*V*_OC_ of all the samples had marginal differences with the highest value at *R* = 0.5 rather than at *R* = 0.6. At *T*_PDA_ = 1000 °C, the reduction of i*V*_OC_ and a different tendency with respect to *R* were observed. The reduced passivation is associated with the damaged tunnel oxide layer, as observed in Fig. 3. The dependency to *R* may be related to the enhanced built-in electric field generated by the high doping ratio around the tunnel barrier. Strong built-in fields improve passivation properties and the transport of carriers across the tunnel oxide.

Next, we explored the passivation properties with respect to *T*_G_ at *R* = 0.5, as shown in Fig. 4b. The thermal treatment improved the passivation as before and a lower *T*_G_ offered a better passivation effect for *T*_PDA_ < 950 °C. The improvement at low growth temperature can be ascribed to the lower activation energy of the B_2_H_6_ decomposition than that of SiH_4_, resulting in a reduced boron concentration during the high temperature deposition^39,40^. As in the case of *R* dependence, the effect of *T*_G_ gradually decreased as *T*_PDA_ was increased, and i*V*_OC_ of the annealed samples at *T*_PDA_ = 950 °C became minimal. At *T*_PDA_ = 1000 °C, the instability of the tunnel oxide layer deteriorated the passivation property and dropped the i*V*_OC_.

Overall, we observed that the growing conditions with respect to *R* and *T*_G_ resulted in different passivation properties at low *T*_PDA_, and the differences became negligible at an optimal *T*_PDA_, which indicated that the crystallization and dopant in-diffusion were more critical to determining the passivation effects within our explored ranges.

### Active boron diffusion and resultant band structures

**Figure 5:**
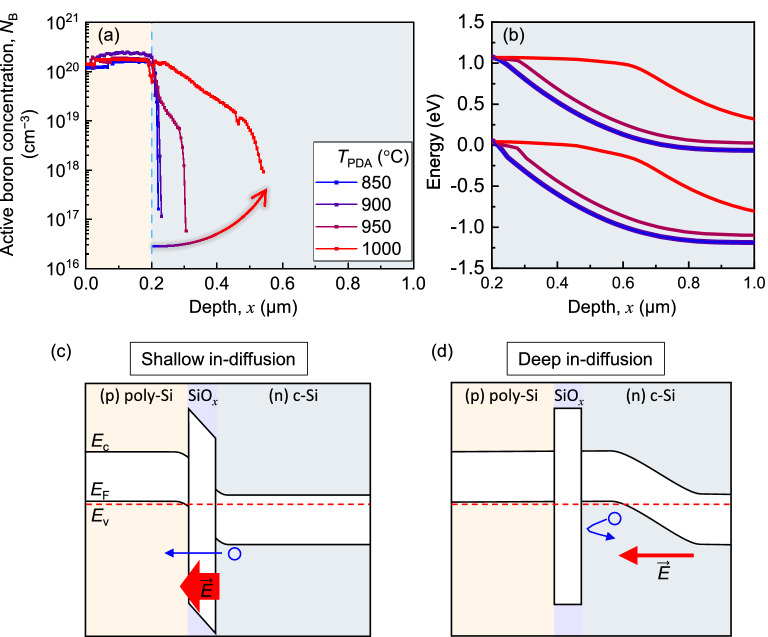
(**a**) ECV profiles of the active boron concentration annealed at 850 °C, 900 °C, 950 °C, and 1000 °C for the samples with 1.5-nm-thick SiO_*x*_ layers and (**b**) simulated band diagrams based on the PC1D simulation. Schematic of the band structures corresponding to (**c**) the shallow in-diffusion and (**d**) deep in-diffusion of the boron dopants.

To determine the correlation between the boron in-diffusion and tunnel oxide, we performed the electrochemical capacitance–voltage (ECV) measurements for the samples with the 1.5-nm-thick oxide layer. The prepared samples were grown at a flow rate of *R* = 0.5 and growth temperature of *T*_G_ = 530 °C, but at different annealing temperatures (*T*_PDA_) in the range of 850–1000 °C. Figure 5a shows the active boron profiles following the thermal annealing. As *T*_PDA_ was increased, the distribution of the boron dopant became more dispersive causing deeper in-diffusion into the c-Si. The enhanced diffusion originated from the enhanced diffusivity caused by the thermal energy and the reduced diffusion barrier owing to the degradation of the oxide layer^18,31^. The passivation property of the selective contact deteriorates at high *T*_PDA_ because of the following three reasons: First, the excessive in-diffusion of boron atoms into the silicon substrate forms an extended p-type region in the n-type substrate, following which the p-type flat-band region across the oxide greatly suppresses the field-effect passivation at the oxide region^30,31^. Second, the high concentration of the diffused boron atoms introduces an elevated Auger recombination rate^17,18^. Third, the increase of the aforementioned unpassivated surface area of the c-Si caused by the damage in SiO_*x*_ also deteriorates the passivation.

To determine the resultant band structure from the dopant profiles, we performed PC1D simulation based on our experimental ECV data (see Fig. S3). Figure 5b shows the *T*_PDA_ dependent real-space dispersion of the electronic band structure. Because our hole-selective contacts were p–n junctions, clear band bending was observable regardless of the *T*_PDA_. The higher *T*_PDA_ pushed away the position of depletion region from the tunnel oxide layer and broadened the p–n junction. This effect reduces the electric fields across the tunnel barrier, and the trapezoidal shape of the tunnel barrier is changed to nearly rectangular by the absence (or weakness) of the electric fields (Fig. 5c,d). As the built-in electric field is expected to support the hole carriers in crossing the tunnel barrier via the direct- or/and defect-assisted tunneling as well as thermionic emission, the carrier selectivity is improved^41^. Therefore, the optimal thermal annealing plays an important role in enhancing the passivation of the hole-selective contacts through the formation of the electric fields across the tunnel barrier and high-quality chemical passivation from the robust tunnel oxide layer.

## Conclusion

In this study, we investigated the effects of thermal annealing on the passivation properties of tunnel oxide passivated hole-selective contacts grown on an n-type c-Si. The samples with 1.5-nm-thick oxide layers showed a higher onset temperature of oxide degradation compared to samples with 1.2-nm-thick oxide layers, which was reflected in the passivation properties and microstructures of the oxide layers. We explored the effects of thermal annealing under different growth conditions of the poly-Si. We observed that the dopant in-diffusion and damage of the oxide layer were the two dominant factors influencing the passivation quality, whereas the growth temperatures of the poly-Si and gas flow rate ratios of the precursor and dopant gases had a relatively weaker impact on the formation of hole-selective contacts. Active boron dopant concentration and the corresponding band structure formed were also investigated, which revealed that the position and gradient of the depletion region played an important role in gaining high carrier selectivity. These findings offer useful information to understand the formation mechanism of hole-selective contacts under thermal annealing and may have potential implications in the designing of crystalline silicon solar cells with high-conversion efficiency.

## Methods

The c-Si wafers used in this study were n-type Czochralski (100)-oriented c-Si wafers. We used 200-μm-thick c-Si with a resistivity of 1.2–1.3 Ω cm and size of 156.7 × 156.7 mm^2^. All wafers were cleaned using deionized (DI) water + H_2_O_2_ + HCl (RCA2) at 85 °C and chemically polished using 10% KOH solution at 80 °C. The wafer surfaces were etched to approximately 10 μm. The wafers were immersed into 10% HF solution for 10 s to remove the native silicon oxide from the wafers.

The cleaned wafers were loaded into the LPCVD chamber and purged using N_2_ gas for 45 min at a temperature of 530 °C. Then, the SiO_*x*_ layers were grown by dry thermal oxidation at a temperature of 630 °C and oxygen flow rate of 3 SLM to form 1.2-nm-thick (1.5-nm-thick) layers under a pressure of 0.3 Torr (760 Torr) for 15 min. The hole-selective layers were grown using the LPCVD method. We fabricated 200-nm-thick B-doped polysilicon on the oxidized wafers with the gas flow ratio, *R* ≡ [B_2_H_6_]/[SiH_4_], in a mixture of B_2_H_6_ and SiH_4_ precursor gases while fixing the flow rate of the SiH_4_ gases at 100 SCCM and a temperature range of 500–550 °C. After the deposition, the samples were exposed to post-deposition annealing at a temperature range of 750–1000 °C using a tube furnace under a nitrogen atmosphere.

After the sample preparation, the i*V*_OC_ values were determined by the QSSPC method (WCT-120, Sinton Instruments). The microstructural images were obtained using TEM (JEOL, JEM-ARM300F) operated at 200 keV. The TEM samples were prepared using a focused-ion beam. Active boron-concentration profiles were obtained using the ECV profiling technique (WEB Wafer Profile CVP21), and a PC1D simulation was performed to describe the band diagrams based on the experimental results.

## Supplementary Information

Supplementary Information.

## Data Availability

The data that support the findings of this study are available from the corresponding authors upon reasonable request.
