# Supplementary material for: Thermal annealing effects on tunnel oxide passivated hole contacts for high-efficiency crystalline silicon solar cells
Source: Sci Rep. 2022 Sep 2;12:15024. doi: 10.1038/s41598-022-18910-5 (PMC9440010; doi:10.1038/s41598-022-18910-5)
Supplement: Supplementary file 1 — Supplementary Information. [file 41598_2022_18910_MOESM1_ESM.docx]

**Supplementary Information**

**Thermal annealing effects on tunnel oxide passivated hole contacts for high-efficiency crystalline silicon solar cells**

Yong-Jin Kim, I Se Kweon, Kwan Hong Min, Sang Hee Lee, Sungjin Choi, Kyung Taek Jeong, Sungeun Park, Hee-eun Song, Min Gu Kang, Ka-Hyun Kim

**Figure S1**. QSSPC results of the Auger-corrected inverse effective lifetime at *T*_PDA_ = 750 °C and *T*_PDA_ = 1000 °C with fitting results.

**Figure S2.** Sheet resistances of the samples annealed at *T*_PDA_ = 850 °C as a function of *R*.

**Figure S3.** Doping concentration profiles from the ECV data (black) and the simulated profiles of electron (blue) and hole (red) concentration for the case of *T*_PDA_ = 1000 °C.
